# Selection of *pfdhfr*/*pfdhps* alleles and declining artesunate/sulphadoxine-pyrimethamine efficacy against *Plasmodium falciparum* eight years after deployment in eastern Sudan

**DOI:** 10.1186/1475-2875-12-255

**Published:** 2013-07-19

**Authors:** Nahla B Gadalla, Tajeldin M Abdallah, Sharanjeet Atwal, Colin J Sutherland, Ishag Adam

**Affiliations:** 1 Department of Immunology and Infection, London School of Hygiene and Tropical Medicine, London, UK; 2 Tropical Medicine Research Institute, National Centre for Research, Khartoum, Sudan; 3 Faculty of Medicine, University of Kassala, Kassala, Sudan; 4 Faculty of Medicine, University of Khartoum, Khartoum, Sudan

**Keywords:** *Plasmodium falciparum*, *pfdhfr*, *Pfdhps*, *Pfcrt*, *Pfmdr1*, Artesunate, Sulphadoxine-pyrimethamine, Sudan

## Abstract

**Background:**

Artesunate/sulphadoxine-pyrimethamine (AS/SP) has been the first-line treatment for falciparum malaria in Sudan since 2004. The impact of this combination on anti-malarial resistance-associated molecular markers has not been investigated. In this study, an evaluation of the efficacy and prevalence of drug resistance alleles (*pfcrt*, *pfmdr1*, *pfdhfr* and *pfdhps*) eight years after the adoption of AS/SP in eastern Sudan is reported.

**Methods:**

A 28-day follow-up efficacy trial of AS/SP was conducted in eastern Sudan during the 2012 transmission season. Blood smears were collected from patients on days 0, 1, 2, 3, 7, 14, 21 and 28. Blood spots on filter paper were obtained pre-treatment and on the day the patient was parasite positive by microscopy. Genotyping of alleles was performed by qPCR (*pfcrt 72*–*76* and *pfmdr1* copy number) and direct sequencing of *pfmdr1*, *pfdhfr* and *pfdhps*.

**Results:**

Sixty-three patients out of 68 (93%) completed the 28-day follow-up, adequate clinical, and parasitological response occurred in 90.5% and 85.3% of the patients in the per-protocol and intent-to-treat analyses, respectively. PCR corrected per-protocol efficacy was 93.7%. The enrolment prevalence of *pfcrt*-CVMNK was 30.2% and *pfmdr1*-N86 was 40.3%. The *pfmdr1* haplotype NFD occurred in 32.8% of pre-treatment samples and was significantly higher than previous reports (Fisher’s exact *p* = 0.0001). The *pfdhfr*-51I/108N combination occurred in all sequenced isolates and 59R was observed in a single individual. *pfdhps* substitutions 436A, 437G, 540E, 581G and 613S were observed at 7.8, 77.3, 76.9%, 33.8% and 0.0%, respectively. Treatment failures were associated with the *pfdhps* haplotype SGEGA at these five codons (OR 7.3; 95% CI 0.65 - 368; *p* = 0.048).

**Conclusion:**

The decrease of CQR associated genotypes reflects the formal policy of complete removal of CQ in Sudan. However, the frequency of markers associated with SP failure is increasing in this study area and may be contributing to the treatment efficacy falling below 90%. Further monitoring of AS/SP efficacy and of post-treatment selection of *pfdhfr* and *pfdhps* alleles *in vivo* is required to inform future treatment guidelines.

## Background

Malaria continues to affect over two million people worldwide with more than 600,000 reported deaths in 2010, most of these in sub-Saharan Africa [1]. There are increasing efforts to control, eliminate or eradicate malaria in many countries [2], however these are hindered by ineffective treatment and vector control strategies that cannot be sustained in economically disadvantageous countries.

Malaria in Sudan causes morbidities and mortality among all age groups and was responsible for more than 95,000 hospital admissions in 2011 [3]. *Plasmodium falciparum* causes over 95% of malaria infections and drug resistance to CQ and SP is well established [4-7]. The wide-scale deployment of artemisinin combination therapy (ACT) across malaria-endemic zones is intended to counteract the emergence and spread of resistance [8]. Artesunate-sulphadoxine/pyrimethamine (AS/SP) and artemether-lumefantrine (AL) became the first- and second-line of treatment, respectively, for uncomplicated *P*. *falciparum* malaria in 2004 [9]. Recently, the efficacy of AS/SP has been questioned among practicing physicians with some patients returning with recurring infections. The *in vivo* efficacy of SP in eastern Sudan was reported at 82% [10], similar to Tanzania (86%) [11] and high levels of resistance to SP in East Africa [12,13] may render the short half-life artesunate partner unprotected. Furthermore the emergence of slow clearing parasites following artesunate treatment in South East Asia [14,15] has prompted closer monitoring of the efficacy of ACT and necessitated extensive research on molecular markers of resistance to artemisinins and partner drugs.

Specific haplotypes of the *pfcrt* gene at positions 72 – 76 (CVMNK; wild haplotype and CVIET; mutant Asian/African haplotype; mutant SVMNT South American haplotype) are well-established markers for resistance to CQ [16,17]. In eastern Sudan the prevalence of the *pfcrt*-76T increased from 54% in the early 1990’s [18,19] to more than 90% in the 2000’s [7,20,21]. However, the *pfcrt*-76K allele has returned to high prevalence in parts of Africa where CQ was discontinued, such as Malawi [22,23], Kenya [24,25], Senegal, [26] Tanzania [27,28] and Mozambique [29].

*Pfmdr1* haplotypes N86, 184F and 1246D have been selected by artemether-lumefantrine in eastern Sudan [30] as well as several parts of Africa [31-34]. These haplotypes were also found to increase the risk of treatment failure with AL [35]. Amplification of *pfmdr1* which is associated with decreased sensitivity to artemisinins in South East Asia [36,37] has recently been observed in eastern Sudan [30] and in neighbouring east African countries [38,39]. Therefore, *pfmdr1* may continue to be under the influence of ACT currently employed in Sudan.

Resistance to pyrimethamine is due to a set of sequential mutations in the dihydrofolate reductase gene (*dhfr*) [40,41]. The serine to asparagine at position 108 is essential with additional mutations at positions N51I, C59R and I164L leading to higher resistance levels [42,43]. Mutations in the *pfdhps* gene (S436A, G437A, K540E, A581G and A613S) encoding the target enzyme for sulphadoxine are also predictors of treatment failure *in vivo*[44]. It has been recommended that SP should be discontinued in areas where the prevalence of the *pfdhps*-540E allele is more than 50% [12]. In eastern Sudan, the prevalence of the *pfdhfr* C**I**C**N**I haplotype at codons 50, 51, 59, 108 and 164 has been reported at 90% [20]. The *pfdhps* haplotype S**GE**AA (436/437/540/581/613) was higher than 80% in the current study area prior to the introduction of AS/SP [20]. These two alleles may be maintained in the population by the strong selection pressure imposed by SP as an ACT component.

In this study, the prevalence of molecular markers in four genes (*pfcrt*, *pfmdr1*, *pfdhfr* and *pfdhps*) associated with drug resistance following the wide scale deployment of AS/SP in eastern Sudan is investigated and an updated clinical efficacy of AS/SP is reported.

## Methods

### Patients enrolment

Patients were recruited between January and March 2012 at Fatima Eldiaaig Health Centre in Kassala City in eastern Sudan, which is characterized by moderate perennial malaria transmission with a peak in January [45]. Febrile patients (auxiliary temperature of ≥37.5°C) from all age groups with microscopically confirmed uncomplicated *P*. *falciparum* mono-infection and a parasite count of a minimum of 1,000 a sexual parasites/μl were asked to participate and those who consented were enrolled. Patients with concomitant illnesses and pregnant women were excluded from the study. A structured questionnaire for socio- demographic information and medical history was completed for each patient by a physician. The study was performed as per the WHO guidelines for anti-malarial drug efficacy surveillance methods [46].

### Sample collection and laboratory diagnosis

Finger prick samples were taken from all participants, thick smears were prepared and stained with 10X Giemsa stain and slides were read under a 100X oil immersion field. Parasite density was obtained by counting asexual parasites against 200 leucocytes and parasite density was calculated assuming an average of 6,000 leucocytes/μl. Slides were read by two experienced microscopists: a slide was considered negative if no parasites were detected in 100 high powerfields. Blood spots were collected on Whatman^®^ No. 3 filter paper, air dried and stored in self-sealing bags for DNA analysis pre-treatment and on the day of treatment failure.

### Treatment and follow up

All participants were given, under observation, artesunate/sulphadoxine-pyrimethamine(Artecospe, Guilin pharmaceutical, China) comprising 4 mg/kg artesunate on days 0–2 as a single daily dose and a single dose of SP (25 mg/kg sulphadoxine, 1.25 mg/kg pyrimethamine) on day 0. For children the dose was adjusted by weight and the tablets were dissolved in water for oral administration. A full dose of the drug was repeated in case of vomiting after 60 minutes and half the dose was repeated if the patient vomited between 30 to 60 minutes after drug administration.

Patients were asked to return to the health centre on days 1, 2, 3, 7, 14, 21 and 28 or if they remained unwell, on each visit axillary temperature was measured and a blood smear was taken. Patients were asked about any adverse effects of the drugs such as nausea, vomiting, abdominal pain, itching and rashes: these symptoms were considered drug induced if they were not reported on enrolment.

Patients who remained microscopy negative for asexual parasitaemia of *P*. *falciparum* throughout the follow up period were considered to have achieved adequate clinical and parasitological response (ACPR). Treatment failure was classified as early treatment failure (ETF), late parasitological failure (LPF) or late clinical failure (LCF) according to the WHO guidelines [46]. Those who failed treatment were given AL (Coartem^®^) as per the Sudanese National Malaria Control Programme guidelines.

### DNA extraction and loci detection

Parasite DNA was extracted from dried filter paper in 96-deep-well plates by the Chelex^®^ extraction method [47] with modifications for 96-well plates [34]. The *pfcrt* loci at codons 72 – 76 were detected by a Taqman assay as described previously [7]. Briefly, 5 μl of DNA were added to the master mix containing 0.3 μM of primers CRTD1 and CRTD2 [48] and 0.1 μM of each probe (crt76-CVMNK, crt76-CVIET and crt76-SVMNT).

Regions of *pfmdr1* encompassing polymorphisms at positions N86Y, Y184F, C1034S, D1042N and D1246Y were amplified with previously reported primers [34]. Polymorphic sites in *pfdhfr* N51I, C59R, S108N, I164L, *pfdhps* A436, A437G, K540E, A581G and A613S were amplified as previously described [49] and directly sequenced.

Amplification of the *pfmdr1* gene was investigated by a qPCR assay as previously described [50] employing the Taqman^®^ Universal Mastermix. DNA from clones 3D7 and Dd2 were employed as controls for single and multiple copy number, respectively. Each sample and control was run in triplicate.

*Msp1* and *msp2* polymorphic markers were amplified as described previously [51] to confirm the origin of recurring parasites in those patients that were microscopically positive during the follow up.

### Ethical considerations

The study received ethical clearance from the Health Research Board at Ministry of Health, Kassala State. Written informed consent was obtained from each patient or child guardian.

### Statistical analysis

The intention-to-treat analysis included all enrolled patients who met the inclusion criteria and took at least one full dose of AS/SP. Patients lost to follow-up or withdrawn from the study were considered to be treatment failures. The per protocol (PP) analysis included data for patients who had completed the follow-up. Those patients lost to the follow-up or who were withdrawn because of protocol violations were excluded from the PP analysis. Clinical data were entered in MS-Excel and analysed in SPSS software (SPSS Inc., Chicago, IL, USA). The means (age, weight, temperature and parasite count) were calculated for all patients. The difference in frequency of haplotypes observed in 2003 [20,52], 2006 [30], and in this study were analysed by Fisher’s exact test (STATA Statistical Software: Release 8.1, 2003, Stata Corp., College Station, TX). Odds ratios were calculated where appropriate. The difference was assumed significant if the *p*-value ≤ 0.05.

## Results

A total of 453 patients who came to the clinic with fever and reporting any malaria-like symptoms were screened for *P*. *falciparum* infection. Sixty-eight patients fulfilled the enrolment criteria and consented to participate in the study, sixty-three (92.6%) of whom completed 28-days of follow up. Patient age, weight, temperature, parasite density and gender are summarized in Table 1. Mean time to resolving clinical symptoms such as fever (axillary temperature above 37°C), malaise and nausea was 2.7 days (range 2 – 7 days) and was not a risk for failing treatment.

**Table 1:** Characteristics of study participants

**Variable**	**Mean [range]**
Age (years)	25.8 [2 – 70]
Weight (Kg)	44.9 [6 – 104]
Temperature °C	38.3 [37.5 – 40.7]
Geometric mean parasite density parasites/μl	4167.1 [1,080 – 200,000]
Sex: female (%) male (%)	44.4% 55. 6%

On day 1; 12.7% (8/63) patients were febrile and 20.6% (13/63) patients were parasitaemic. However, all patients cleared their parasitaemia by day 3 with one patient remaining febrile (temperature 38°C). Gametocytaemia was observed in 3.2% (2/63) of patients at recruitment. Adverse effects of the drugs such as nausea, headache, cough, dizziness, abdominal pain and diarrhoea were reported in 34.9% (22/63) of patients between day 19 and 28. In the intent-to-treat analysis where all patients who were enrolled were included in the analysis, the efficacy of AS/SP was 85.3%. While in the per-protocol analysis the efficacy was 90.5%. Two patients failed treatment at day 21, one failed treatment at day 26 and two failed treatment at day 28. The PCR corrected efficacy was 93.6% where 4/5 of treatment failures were classified as recrudescing parasites. Factors such as pre-treatment parasite density, axillary temperature and age did not differ significantly between those who failed treatment and those who were successfully treated.

### Expansion of wild type *pfcrt* and *pfmdr1* haplotypes following withdrawal of CQ

**Figure 1:**
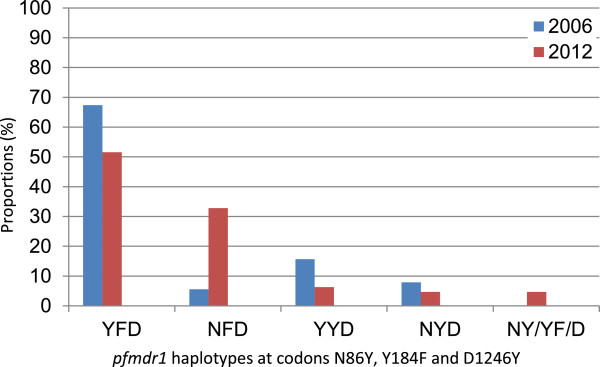
**Difference in proportions of *pfmdr1 *haplotypes in eastern Sudan between 2006 and 2012.**

The wild type *pfcrt* haplotype CVMNK has significantly recovered from 10% in 2003 [52] to 30.2% (Fishers exact *p* < 0.001) in this study. . The N86 allele has also increased significantly from 14% [52] to 40.3% (Fisher’s exact *p* = 0.0059). The prevalence of the wild type Y184 was 15.6% but this was not significantly different from 2006 (Fisher’s exact *p* = 0.16), while there was a significant decrease in mutant 1246Y (Fisher’s exact *p* = 0.018) (Table 2) [30]. *Pfmdr1* haplotypes at positions 86/184/1246 were less diverse than 2006. The major *pfmdr1* haplotype in this study was YFD observed in 51.6% of pre-treatment samples. NFD has significantly increased in prevalence from 5.6% in 2006 to 32.8% in 2012 (Fishers exact *p* = 0.0001) (Figure 1). The NFD haplotype was detected in 3/5 post-treatment samples and 2 samples were YFD. All the isolates analysed in this study carried the wild type alleles at positions S1034 and N1042 and a single copy of the *pfmdr1* gene (mean 1.2; range 0.6 to 1.5).

**Table 2:** **Changes in pre**-**treatment frequencies of *pfcrt *and *pfmdr1 *haplotypes between 2003 and 2012 in eastern Sudan**

***pfcrt/pfmdr1 *allele**	**2003 %**	**2006 %**	**2012 %**	***p*-value 2003/2006**
**CVIET**	93[20] 90[52]		74.6	**0.000/**0.080
**CVMNK**	10[52]		30.2	**0.010**
**CVMNK**/**CVIET**	24[52]		4.8	**0.012**
**N86**	14[52]	12.1[30]	40.3	**0.006/0.0001**
**86Y**	84[52]	85[30]	55.2	**0.006/0.000**
**86 N**/**Y**	2[52]	3[30]	4.5	0.629/0.691
**184F**		74[30]	80.6	0.165
**184 Y**/**F**		0[30]	4.5	0.074
**1246Y**		6[30]	0.0	0.755
**1246 D**/**Y**		3[30]	0.0	0.270

*p*-values were calculated by comparing actual number of patients displaying each genotype from previous studies performed in Gedarif in 2003 [20,52] and Gedarif and Kassala 2006 [30]) and the current study (Kassala) by Fishers exact test.

### Changes in antifolate resistance markers

For the *pfdhfr* gene a total of 59 samples were successfully sequenced for positions 50 to 164. All of which carried the 51I and 108N mutant alleles. This is a significant increase from previous years (Fisher’s exact *p* = 0.041). One sample (1.7%) harboured the mutant 59R and all of the samples were wild-type at position 164. Thus the predominant haplotype for *pfdhfr* in eastern Sudan is 51I/C59/108N.

A total of 65 pre-treatment samples were successfully genotyped at the *pfdhps* positions 430 to 613. A novel polymorphism at position L516F was detected in one sample. The prevalence of the 581G allele significantly increased since the 2003 study from 14% to 33.8% (Fisher’s exact *p* = 0.0005), while the 437G decreased slightly from 89% to 77% (Fisher’s exact *p* = 0.0134) [20]. In pre-treatment samples, the 581G allele was strongly linked to the 540E (*p* = 0.0009) and the 437G (*p* = 0.0018). The prevalence of S436A, A437G, L516F, K540E and A613S are listed in Table 3.

**Table 3:** **Prevalence of *pfdhfr *and *pfdhps *alleles in Sudan before and after adoption of ACT**

***pfdhfr/pfdhps *allele**	**2003 %**	**2012 %**	***p-*value [**[20]**,**[52]**]**
**51I**/**108N**	90[20] 84[52]	100	**0.004/0.001**
**59R**	1[20] 0[52]	1.7	0.477/1.000
**436A**	0[52]	7.8	0.069
**437G**	89[20] 0[52]	77.3	**0.013/0.000**
**516F**		1.7*	
**K540**	20[52]	21.5	**0**.**000**
**540E**	79[20]	76.9	0.860
**540K**/**E**	80[52]	1.5	**0.000**
**581G**	14[20] 10[52]	33.8	**0.001/0.007**
**581A**/**G**	10[52]	1.5	0.202
**A613**	100[20,52]	100	1.000

*p*-values were calculated by comparing actual number of patients displaying each genotype from previous studies performed in Gedarif in 2003 [20,52] and Gedarif and Kassala, 2006 [30]) and the current study (Kassala) by Fishers exact test. * not previously reported.

**Figure 2:**
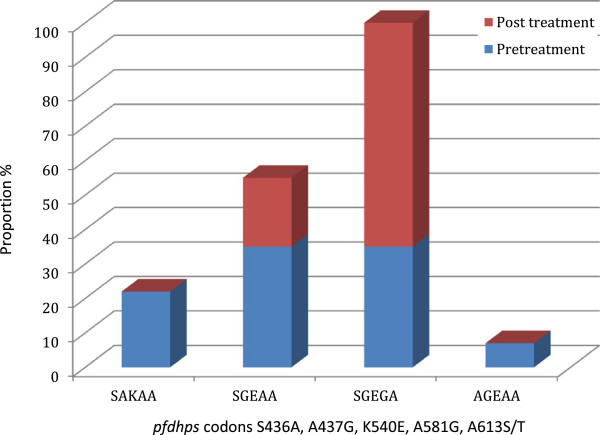
**Proportions of *pfdhps *haplotypes before and after treatment with AS/SP in Kassala State 2012.**

*Pfdhps* sequencing was successful for all five treatment failures, collected at day 21 (2), day 26 (1) and day 28 (2). The haplotype across the five polymorphic codons 436, 437, 540, 581 and 613 are shown for these two groups in Figure 2. Whereas all five samples collected at the time of treatment failure carried the 540E substitution as expected, all of them also carried the 437G and 581G. Thus, the *pfdhps* haplotype SGEGA, at these five polymorphic positions, was significantly more common in parasites detected after treatment (OR 7.3; 95% CI 0.65 - 368; *p* = 0.048) whether these parasites were recrudescing or re-infecting parasites. Further, the presence of this *pfdhps* genotype in the pre-treatment infection was a significant risk factor for later treatment failure (relative risk 7.3, 95% CI 0.87 - 61.6; *p* = 0.030) the wide confidence intervals indicate that these observations need detailed exploration in larger studies.

## Discussion

*Plasmodium falciparum* carrying mutations associated with drug resistance may be less fit to survive in the absence of drug pressure. The continuous use of CQ monotherapy for decades has lead to almost saturation of the *P*. *falciparum* population in eastern Sudan carrying the mutant *pfcrt* allele CVIET [7]. Lower drug pressure is reflected by more wild type *pfcrt* where transmission is higher and perennial in central Sudan [53]. Moreover SP has been used intensively in patients where CQ failed. This has resulted in the high prevalence of the *pfdhfr*/*pfdhps* mutants (51I/108N/437G/540E) in the late 1990’s and early 2000’s [18,20,54]. The adoption of AS/SP in eastern Sudan in 2004 has lead to considerable decrease in malaria cases in the country [9,55-57]. The clinical efficacy of SP monotherapy in Sudan was approximately 68% prior to introduction of AS/SP [58]. Therefore, the wisdom of deploying this SP containing combination has been debated among the malaria research community in Sudan e.g. [59]. The base-line efficacy of AS/SP in eastern Sudan has been reported between 98 - 100% [56,60-62], while we have reported a base-line PCR corrected efficacy of 93% [57]. In the present study the *in vivo* per-protocol PCR corrected efficacy of AS/SP over 28 days is 90.5%. All patients cleared their microscopic parasitaemia by day 3. Approximately 10% of patients exhibited recrudescing parasites as confirmed by *msp1* and *msp2* genotyping during the study. It is important to emphasize the relatively lower transmission season in eastern Sudan compared to other parts of East Africa. Therefore, to definitely establish whether these post-treatment infections were true recrudescence or re-infections requires genotyping at more polymorphic loci.

A limitation to our data is the lack of parallel PCR data for days 3 and 14. Sub-microscopic parasites at these time points have been detected in previous studies from eastern Sudan [7,30]. Detection of parasites on day 3 is an early warning of slow clearance by artemisinin derivatives observed in South East Asia [14,15]. A decline in the efficacy of ACT e.g. AL and DHA/PIP has been reported from Kenya [63]. Sub-microscopic parasitaemia has previously been observed following AL treatment in eastern Sudan [30]. While an artemisinin resistant phenotype has not been reported in Africa [64], the history of anti-malarial resistance suggests that artemisinin resistance is likely to spread to East Africa despite the global efforts in its containment.

In the present study, the prevalence of molecular markers eight years after the adoption of ACT is compared to previous reports from the same study area of eastern Sudan. In this area IPTp is recommended for all pregnant women, as in other parts of Sudan.

The prevalence of a double mutant *pfdhfr* allele (51I/108N) has reached 100%, which clearly demonstrates the effect of drug pressure on this locus. The observation that contrasts with other African countries is the slow evolution of *pfdhfr* in this region where the double mutant 51–108 allele has widely spread while the wild type C59 has been maintained. However it is in agreement with a recent report from Yemen [65]. The 59R is associated with increased resistance to pyrimethamine, therefore absence of this mutation may be a good indicator for the *in vivo* efficacy of SP in this region. The *pfdhfr* 59R has been previously reported at low frequency in Khartoum, central Sudan in 1996/1997 [66] and Nuba mountains in western Sudan in 2003 [67]. However the 51I/108N haplotype has been observed at low frequencies in Kenya and Cameroon in the early 2000s where the *pfdhfr*-51I/59R/108N haplotype currently predominates [68,69]. These studies employed microsatellite markers which demonstrated the divergence of origin of the double (51I/108N) and triple mutants within the same geographic location. An earlier study in eastern Sudan has shown that microsatellites around *pfdhfr* were stable over four consecutive seasons [18]. Thus, the *pfdhfr* allele 51I/108N may have been imported into this area earlier and has been maintained within a limited parasite population in a low transmission area of eastern Sudan. However, it would be of interest to examine *pfdhfr* sequences and microsatellite markers from other regions of Sudan with different transmission intensities to test this hypothesis.

Interestingly in this study, a small decrease in *pfdhps*-437G (Fisher’s exact *p* = 0.0134) is observed compared to previous reports [20]. The 581G allele has significantly increased to 33% and has previously been associated with SP failure in eastern Sudan [20] emphasizing the importance of close monitoring of the parasite population in this region. For the first time, a particular *pfdhps* allele, SGEGA, is significantly associated with treatment failure in patients receiving AS/SP for clinical malaria. However of 23 pretreatment infections harbouring SGEGA, only four recrudesced. The increase of SGEGA has been previously reported from Tanzania in cross sectional surveys following several years of SP use [27]. The potential of this haplotype as a single marker of drug efficacy for this anti-malarial combination should be further investigated.

As previously reported from other African countries [22,24,28], there is a significant decrease in CQR associated genotypes *pfcrt*-76T/*pfmdr1*-86Y. Wild type genotypes at these two loci have significantly recovered, which provides supporting evidence that CQ use has been successfully reduced [70]. Interestingly the multiple copy *pfmdr1* genotype previously reported from eastern Sudan [30] was not observed here. This genotype is associated with resistance to mefloquine (MQ) in South East Asia [50] and may not spread in the absence of MQ pressure, which is the case in Sudan. In addition *pfmdr1*amplification has failed to be established and spread in Africa, possibly due to lack of the use of MQ across the continent.

In Sudan SP is recommended for iPTp and the availability of SP as an over-the-counter drug maintains the pressure on the parasite population. The efficacy of AS/SP is reaching the current threshold for anti-malarial policy change of 90%. Continuous use of SP as a partner to artesunate may be the cause of declined AS/P efficacy. In addition discontinuation of SP in areas where the population prevalence of *pfdhps*-540E is above 50% is also recommended [12,71]. Monitoring of SP resistance associated loci, and in particular the SGEGA *pfdhps* haplotype identified here, is required to provide informed treatment guidelines in the near future. In addition the emergence of slow parasite clearance in South East Asia warrants close monitoring of the efficacy of ACT and molecular markers associated with resistance to partner drugs.

## Competing interests

The authors declare that they have no competing interests.

## Authors’ contributions

NBG carried out the molecular genetic studies, the sequence alignment, analysed the results and drafted the manuscript. TMA carried out the *in vivo* study, analysed the clinical data and drafted the manuscript. SA participated in the molecular genetic studies and analyses. CJS designed the molecular study, performed the statistical association analysis, contributed to the manuscript and critically revised it, IA conceived of the study, participated in its design and coordination and drafted the manuscript. All authors read and approved the final manuscript.
